# Spiking Neural Models of Neurons and Networks for Perception, Learning, Cognition, and Navigation: A Review

**DOI:** 10.3390/brainsci15080870

**Published:** 2025-08-15

**Authors:** Stephen Grossberg

**Affiliations:** Departments of Mathematics and Statistics, Psychological and Brain Sciences, and Biomedical Engineering, Boston University, Boston, MA 02215, USA; steve@bu.edu

**Keywords:** neural network, brain, learning, spiking neuron, development, perception, cognition, emotion, navigation, spatial pattern

## Abstract

This article reviews and synthesizes highlights of the history of neural models of rate-based and spiking neural networks. It explains that theoretical and experimental results about how *all* rate-based neural network models, whose cells obey the membrane equations of neurophysiology, also called shunting laws, can be converted into spiking neural network models without any loss of explanatory power, and often with gains in explanatory power. These results are relevant to all the main brain processes, including individual neurons and networks for perception, learning, cognition, and navigation. The results build upon the hypothesis that the functional units of brain processes are spatial patterns of cell activities, or short-term-memory (STM) traces, and spatial patterns of learned adaptive weights, or long-term-memory (LTM) patterns. It is also shown how spatial patterns that are learned by spiking neurons during childhood can be preserved even as the child’s brain grows and deforms while it develops towards adulthood. Indeed, this property of spatiotemporal self-similarity may be one of the most powerful properties that individual spiking neurons contribute to the development of large-scale neural networks and architectures throughout life.

## 1. Introduction: Early Contributions to AI and Neural Networks—Two Separate Fields

In 1957, when I was a Freshman at Dartmouth College taking introductory psychology, I introduced the then-revolutionary neural network paradigm of using systems of nonlinear differential equations to link brain mechanisms to psychological behaviors, as well as the equations for cell activations, or short-term memory (STM) traces; activity-dependent habituation, or medium-term memory (MTM) traces; and learning and memory, or long-term memory (LTM) traces that are still widely used today in biological neural network models—See https://en.wikipedia.org/wiki/Stephen_Grossberg.

As is the case with many scientific pioneers, so far as I knew, there was no field of neural networks when I made my first discoveries. I certainly did not know anyone else who was studying them. It was not until quite a few years later that I heard about the McCulloch–Pitts neuron model [1] and met Warren McCulloch at MIT shortly after I became an assistant professor there in 1967.

**Figure 1:**
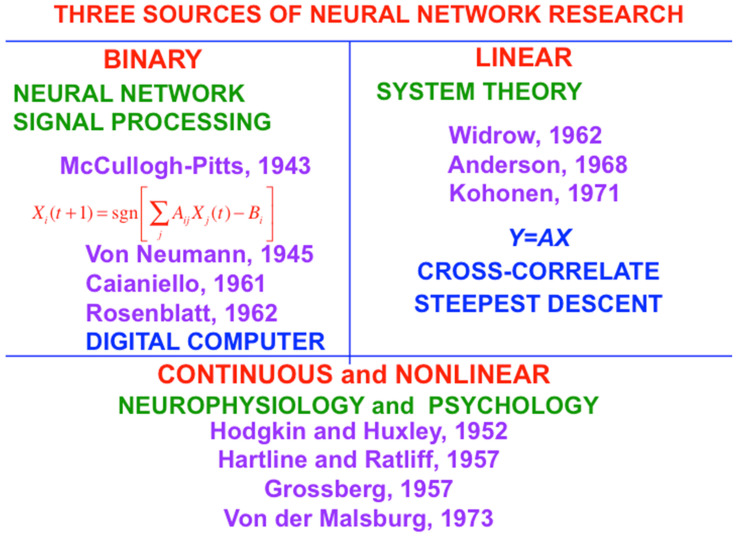
Three sources of neural network research: Binary, linear, and continuous and nonlinear. My own work has contributed primarily to the third. [Reprinted with permission from Grossberg (2021)] [2].

My ignorance was probably useful to me because the binary-threshold neuron that McCulloch and Pitts introduced was unable to model the analog nature of brain dynamics, although it did inspire the great John von Neumann to introduce the digital computer. Figure 1 summarizes three sources of neural network research. This figure contrasts the binary neuronal models of scientists like McCulloch, Pitts, and von Neumann with the continuous and nonlinear neuronal models that I introduced and began to develop in order to eventually provide unified and principled explanations of hundreds of psychological and neurobiological experiments, including *how*, *where* in our brains, and *why* from a deep computational perspective, we can consciously see, hear, feel, and know things about a changing world that is filled with unexpected events, and use our conscious states to plan and act to realize valued goals.

My explanation of how our conscious states enable goal-oriented actions is my proposed solution of the classical Mind-Body Problem. My 2021 Magnum Opus, *Conscious Mind*, *Resonant Brain: How Each Brain Makes a Mind* [2], provides a self-contained and non-technical overview and synthesis of these models, and the interdisciplinary experimental data that they explain.

It was also good luck that I did not know that, in 1956, the year before I came to Dartmouth as a Freshman, there was a Dartmouth Summer Research Project on Artificial Intelligence, attended by many of the most famous practitioners of early Artificial Intelligence, or AI, including John McCarthy, Marvin Minsky, Allen Newell, Oliver Selfridge, Claude Shannon, and Herbert Simon.

I was lucky to be ignorant of this activity, because the primary outputs of this conference were symbolic models of intelligence that could not explain properties of biological intelligence that are emergent, or interactive, properties of interactions among many brain regions. My own work has pioneered and developed such explanations, often with the collaboration of over 100 gifted PhD students, postdoctoral fellows, and faculty in the Center for Adaptive Systems, the Graduate Program in Cognitive and Neural Systems, and the Department of Cognitive and Neural Systems that I founded at Boston University in 1982, 1988, and 1991, respectively. For a brief history of my efforts until 2010 *Towards Building a Neural Networks Community*, see https://sites.bu.edu/steveg/files/2016/06/GrossbergNNeditorial2010.pdf.

This history emphasizes that symbolic AI and neural networks started as two parallel and often non-interacting fields. It was only years later that symbolic AI hit a brick wall, and its practitioners began to look to neural network models to overcome that bottleneck. By that point, scientists like me had already established the field of neural networks and many of its foundational models. Unfortunately, the Johnny-come-lately neural network practitioners frequently failed to cite prior published work by other authors, which was at that time all too familiar behavior by MIT AI authors.

Marvin Minsky was one of the MIT scientists who eventually realized that he had to move from symbolic AI to neural networks. Marvin and I were both in the MIT mathematics department for a number of years, and he attacked my early work in neural networks as part of his symbolic AI marketing blitz. His ultimate move from symbolic AI to neural networks is illustrated by his 1986 book *Society of Mind* [3]. By then, however, Marvin’s book was derivative to the many archival articles that my colleagues and I had already published.

One anecdote about Marvin still brings a smile to my face. After I founded the International Neural Network Society (INNS) and its archival journal *Neural Networks* in 1988, INNS used to hold annual international conferences on neural networks, the first one in Boston in 1988; see https://apps.dtic.mil/sti/html/tr/ADA230283/index.html.

I had earlier, in 1987, been the General Chairman of the first IEEE International Conference on Neural Networks (ICNN’87) that was held in San Diego. As I wrote in the above attachment from 2010, “The budget for ICNN’87 program development was basically nil, and various people at IEEE expressed their doubts that ICNN could be brought off at all. In order to get the word out, Gail [Carpenter] and I put together the conference brochure and program, stuffed thousands of brochures into envelopes, and went to the post office near our home in Newton Highlands, MA, on the day before Christmas in 1986 to have the post office put stamps on the envelopes. Our request was denied, so we spent that afternoon licking stamps ‘for the cause’. As it turned out, ICNN’87 was a brilliant success with almost 2000 people in attendance.”

The INNS and IEEE conferences eventually merged to become the annual International Joint Conference on Neural Networks (IJCNN), which remains the largest international conference on neural networks to the present time; see https://www.inns.org/ijcnn-home. I am proud to have had the opportunity to contribute to the formation and development of these conferences, as well as various other founding infrastructure for the fledgling field of neural networks.

I was General Chairman of one of these conferences, and arranged for Marvin Minsky to give a plenary lecture, in keeping with my lifelong belief in bringing together multiple perspectives to energize interdisciplinary scientific education and research. Unfortunately, Marvin had such a high opinion of himself that he rarely prepared his lectures beforehand. Apart from the fact that Marvin spent most of his time telling jokes, he said one thing that I will never forget. He began his talk by saying that he made one big mistake during his career: “I underestimated Grossberg”. Suffice it to say, the audience roared its approval.

## 2. Why Neural Networks? Early Inspiration from Human Verbal Learning Data

My first modeling discovery was inspired by psychological data about verbal learning by humans. One classical method for studying verbal learning is called the *serial anticipation method*, whereby a human learner predicts the next item in a list before it appears, then the item after that, and so on for enough learning trials until the entire list can be predicted without errors on a couple of runs through the list.

One might initially think that this kind of learning is of limited interest. In fact, the learning of verbal lists presents us with many of the same basic problems that need to be solved to better understand all phenomena about serial order in behavior [4], including language, musical performance, motor skills, or spatial navigation in a complex environment.

**Figure 2:**
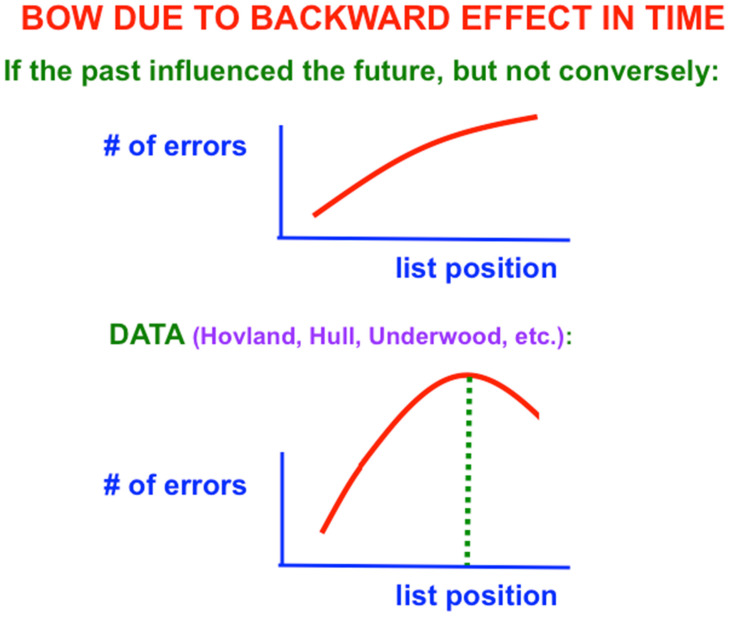
(**top panel**) Monotone increasing errors at increasing list position if the past influenced the future, but not conversely. (**bottom panel**) A bowed serial position curve occurs when “the future influences the past” during serial learning. [Reprinted with permission from Grossberg (2021)] [2].

**Figure 3:**
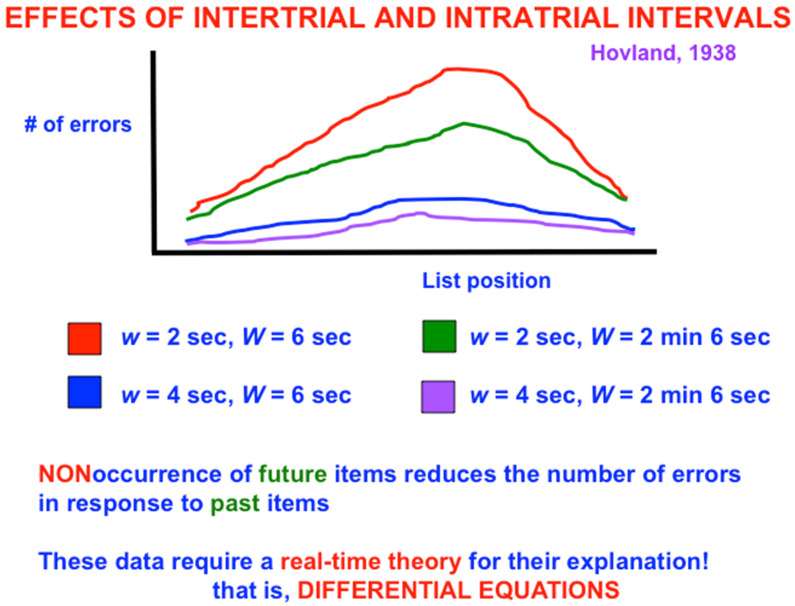
Data showing the bowed serial position curve. This kind of data emphasizes the importance of modeling how our brains give rise to our minds using nonlinear systems of differential equations. [Reprinted with permission from Grossberg (2021)] [2].

One might also initially think that learning of verbal lists is easy and boring. After all, as one tries to predict the next item when one gets deeper into the list, more errors should occur because there is more response interference from the previous items. The number of errors should increase from the beginning to the end of the list (Figure 2, top panel). Right? Actually, Wrong: The middle of the list is hardest to learn, with the beginning and end of the list easier to learn (Figure 2, bottom panel; and Figure 3). This *bowed serial position effect* had resisted explanation for years after it was studied by great American psychologists like Carl Hovland [5,6,7].

As Figure 2 (top panel) indicates, errors should increase from the beginning to the end of the list if only the learning of previous, or past, items in the list influenced learning of future items. What causes the bow is the fact that the rest interval between successive presentations of the list (called the *intertrial interval*) is longer than the presentation rate of items within the list (called the *intratrial interval*). In other words, the *non-occurrence* of items during the rest interval *after* the list items are presented makes it easier to remember learning of associations between previously presented items near the end of the list. This is a *backward effect in time* that occurs when *the future influences the past*. These data excited me a lot and drove me in 1957 to introduce neural networks with STM and LTM traces.

Figure 3 shows a schematic of serial learning curves for which, when the intratrial interval is 2 s, increasing the intertrial interval W from 6 s (red curve) to 2 min and 6 s (green curve) causes the number of errors in the middle of the list to collapse considerably. The same is true when the intratrial interval is 4 s and the intertrial interval increases from 6 s (blue curve) to 2 min and 6 s (purple curve). The latter pair of curves has fewer overall learning errors than the former pair because the slower presentation rate enables previously activated cell populations to decay more, and thus to interfere less, with current learning.

Because it takes a while for STM traces to decay, LTM associations could be learned between multiple pairs of list items. The intuitive reason for the bowed serial position curve is that, as items are presented through time, there are more cells that are simultaneously active as one gets deeper into the list. More simultaneously active STM traces at later list items causes the spatial distribution of large LTM traces across the network to also increase, thereby causing more response interference in the middle, than the beginning, of the list, and correspondingly more errors during learning of the middle than the beginning. During the intertrial interval, however, no additional items are presented, so no more STM traces are activated. As a result, fewer large LTM traces are learned near the end of the list, and thus there is less response interference and fewer errors during learning.

My neural model to explain these data used a recurrent associative neural network in which every letter is represented by a node (actually, a learned recognition category), and pairs of adaptive connections occur between each pair of nodes, so that any pair of items can, in either order, be associated.

Why pairs of connections are needed is illustrated by the simplest example of *backward learning*: If, say, you practice the list AB several times until, in response to A you can guess the answer B with high probability, then it is also the case that presenting B leads to guessing A with higher probability as well. Thus, there must be an adaptive connection from node A to node B *and* from node B to node A.

These examples drove me to introduce the paradigm of neural networks to explain how recurrent neural networks with adaptive weights between every pair of nodes could simulate the bowed serial position curve in response to multiple presentation and rest intervals.

## 3. How I Realized that My Model Is a *Neural* Network: Thinking in Real Time

**Figure 4:**
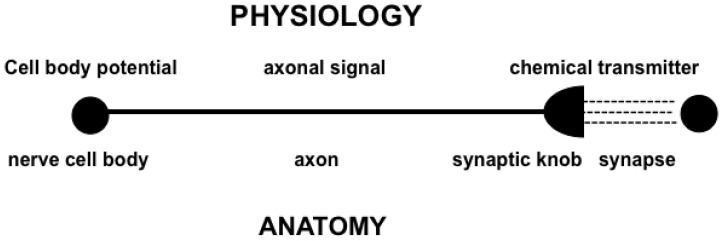
Some anatomical and physiological properties of individual neurons. [Reprinted with permission from Grossberg (2021)] [2].

When I first did the above work, I knew nothing about neuroscience. I was driven to my networks entirely by a psychological analysis of the dynamics of serial and paired associate learning by human learners. It was only when I talked to premedical student friends of mine who were learning about the brain that I realized my model networks were *neural* networks, because the model neurons of my networks mimicked what they were learning about neurons in the brain (Figure 4), including the existence of cell bodies and their STM activities, or potentials; STM-activated signals moving along network connections, or axons; and adaptive weights, or LTM traces, that are computed in synaptic knobs at the ends of axons, where they could learn associations between their signals and the STM traces at abutting cell bodies. The LTM traces were interpreted as the chemical transmitters that are produced in the synaptic knobs and released into the synapses between cells, thereby activating the STM traces, or potentials, of abutting postsynaptic cells.

I like to summarize why a psychological analysis can lead to a model of brain learning by saying that: “Brain evolution needs to achieve behavioral success”. This is just another way to say that Darwinian Selection of our brains evolves brains that *can* achieve behavioral success, if only because successful behaviors are crucial to survival of both individuals and species.

It is also worth noting that my psychological analyses led to neural models of brain dynamics, including learning, because I thought about how the behavior of individuals *autonomously adapts in real time* to world that is filled with unexpected events. As I wrote in Grossberg (2021) [2], “Knowing how to do this is currently an art form. There are no algorithms at present that can assure success in this endeavor. It requires that one be able to translate piles of static data curves into hypotheses about an underlying mental process that is evolving continuously in time within each individual mind. Doing this well requires that one develops intuition into the functional meaning that is hidden in these piles of static data curves and has enough modeling training to express one’s intuitions with appropriate mathematical concepts and equations.… In other words, brain networks define a natural computational embodiment of design principles whereby individuals can adapt on their own to a changing world, and this is why brain anatomies look the way that they do. Models of such brain networks naturally incorporate the types of nonlinear, nonlocal, and nonstationary processes that scientists like Helmholtz, Maxwell, and Mach had already begun to realize are essential.”

Currently popular neural network models like back propagation and Deep Learning, among their other computational weaknesses, cannot autonomously learn about a changing world. See Grossberg (1988, 2020) [8,9] for discussion of 17 computational weaknesses that these models have which do not occur in biological neural models like Adaptive Resonance Theory, or ART.

It took me ten years to start getting my modeling results published. That is because no one else was doing neural network modeling at the time, and there were no journals dedicated to publishing such models. My model of serial learning and the bowed serial position effect got published in Grossberg (1969c) [10] and Grossberg and Pepe (1970, 1971) [11,12], where James Pepe was my first PhD student after I became an assistant professor of applied mathematics at MIT in 1967.

## 4. The Functional Units of STM and LTM Are Spatial Patterns, Not Spikes

The example of serial learning illustrates the fact that the functional units that govern behavioral success are *distributed patterns* on the network and system levels, not single spikes, or sequences or bursts of spikes, in individual neurons. During serial learning, evolving spatial patterns of STM traces are sampled and learned by evolving spatial patterns of LTM traces. This raises the question of why so many brain neurons use spikes to send signals down their axons. I will propose a multi-faceted answer to this question below.

This realization that spatial patterns of STM and LTM are the functional units of brain networks led me to prove global limit and oscillation theorems about the largest classes of neural networks in which spatial patterns of STM traces drive learning of spatial patterns of LTM traces. For example, a spatial pattern of STM traces could be activated by a pattern of feature detectors that represents a visually perceived object, or by an active learned top-down expectation that reads out the feature pattern that its recognition category has earlier learned, or by a motor command that activates a synergy of muscles that contract synchronously in equal time to reach an object.

I was luckily able to publish these articles between 1967 and 1971 in prestigious journals like the *Proceedings of the National Academy of Sciences* and the *Bulletin of the American Mathematical Society* [13,14,15,16,17,18,19,20,21] because Norman Levinson, who was a famous mathematician, MIT Institute Professor, and member of the National Academy of Sciences who made significant contributions to the field of nonlinear differential equations—the formalism that expresses my neural models—believed in my work and submitted several of my articles. Norman was the most famous student of Norbert Weiner, the father of Cybernetics, so was sympathetic to mathematical results aimed at better understanding biological intelligence. I take the liberty of calling him Norman because he and his wife Zipporah (“Fagi”) Levinson adapted me as a kind of scientific son and, among other generous gestures, let me accompany them when they went on sight-seeing trips during mathematical conferences that we attended.

## 5. From Spatial Pattern Learning to Space–Time Pattern Learning: Avalanches

Having mathematically analyzed general conditions under which spatial patterns of STM and LTM could be learned, I then studied how arbitrarily complicated space–time patterns could be learned and performed [22,23]. I first asked: What is the simplest network that can learn to perform an arbitrarily complicated sequence of patterns or actions, such as a piano sonata, dance, or other skilled sequence? What is the minimum number of cells needed to do this? I soon realized that the answer is ONE!

This insight is compatible with the fact that small brains, with relatively few neurons, can carry out complex reflexes. For example, *Wikipedia* summarizes that the brain of a medicinal leech contains 10,000 neurons, of a lobster contains 100,000 neurons, and of an ant contains 250,000 neurons. In contrast, human brains contain 86,000,000,000 neurons. If small brains can learn and perform arbitrarily complex space–time patterns, then why do humans need such big brains? What do the extra neurons do?

**Figure 5:**
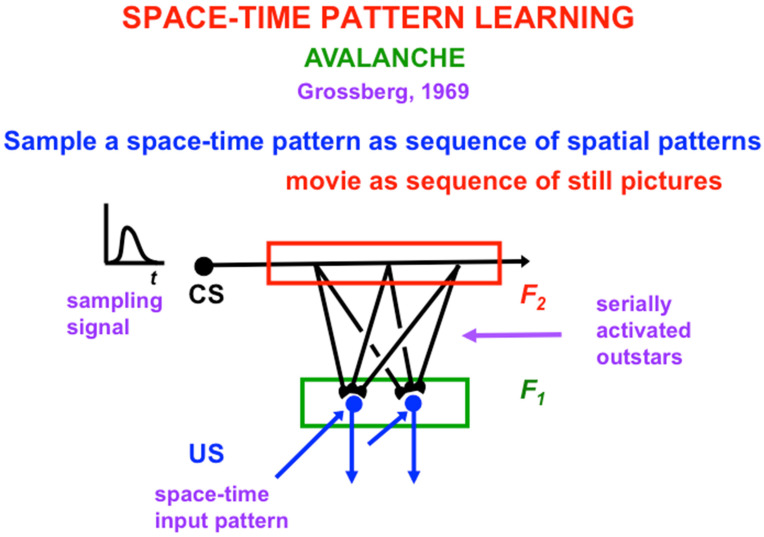
A single avalanche sampling cell can learn an arbitrary space-time pattern by sampling it as a temporally ordered series of spatial patterns using a series of outstars. Once an avalanche’s sampling cell starts to fire, there is no way to stop it from performing the entire space–time pattern, no matter how dire the consequences. [Reprinted with permission from Grossberg (2021)] [2].

I called the network with just a single neuron an *avalanche* because it learns and performs an arbitrarily complicated space–time pattern as a linearly ordered sequence of spatial patterns (Figure 5). An avalanche has the serious disadvantage of being insensitive to environmental feedback. I liked to say that, if a concert pianist learned the Appassionata Sonata of Beethoven using an avalanche and was performing it in a concert hall when a fire broke out, the pianist would be forced to complete playing the sonata even as the stage, piano, and his or her person began to be consumed by the flames. Grossberg (2021) [2] provides a systematic evolutionary explanation of how many more neurons are used in neural networks and architectures that are sensitive to different kinds of environmental feedback, leading to the complexity of the human brain.

## 6. Equations, Modules, and Modal Architectures Model How Brains Make Minds

Just as in mathematical physics, my neural models are expressed using a few basic mathematical equations that are analogous to Newton’s laws for celestial mechanics. Also like in physics, my models use a somewhat larger number of microcircuits, or modules, that are defined using these equations and that are analogous to physical models of atoms and molecules. Finally, these equations and microcircuits are specialized and combined in a variety of ways to create neural architectures that are analogous to macroscopic objects in the external world.

I have called these architectures *modal architectures* because they help to model different modalities of intelligence, including vision and visual object recognition; audition, speech, and language; development; attentive learning and memory; cognitive information processing and social cognition; reinforcement learning and motivation; cognitive–emotional interactions, including reinforcement learning; navigation; cognitive and motor planning; sensory–motor control and robotics; and mental disorders such as Alzheimer’s disease, autism, medial temporal amnesia, schizophrenia, ADHD, PTSD, auditory and visual agnosia and neglect, and disorders of slow wave sleep. These models involve many parts of the brain, ranging from perception to action, and multiple levels of brain organization, ranging from individual spikes and their synchronization to cognition.

## 7. Additive and Shunting Rate-Based Neural Models and the Noise-Saturation Dilemma

For many years after I introduced neural networks in 1957, all my models were described by *rate-based differential equations*. Differential equations are used as the mathematical formalism in many scientific disciplines, including physics, because they describe how events evolve in time. The term “differential” describes the rates, or “differentials,” at which processes evolve in time. I discovered that just a few different types of differential equations, indexed to keep track of what neurons they are describing, could naturally describe the dynamics of huge numbers of individual neurons, how they interact in neural networks, and the behavioral properties that emerge from these interactions. In fact, such rate-based neural networks were used to successfully model all the equations, modules, and modal architectures that I used to provide principled explanations of all the kinds of psychological processes that I listed above, including quantitative computer simulations of the data from hundreds of psychological and neurobiological experiments.

The success of these explanations using rate-based models raised the question: If rate-based neural models can provide explanations and quantitative simulations of so much data, then when are spiking models needed? I will return to this issue below.

But first, let me note that the rate-based STM activity of a cell body models the continuously varying potential of the cell, which is an average of many factors that influence the cell. The rate-based STM-activated signal along an axon is an average of many individual spikes that travel down the axon. The rate-based LTM trace is also an average of many factors operating on a finer spatial and temporal scale.

How to define such averages in rate-based models had long concerned me. My article Grossberg (1969e) [24] on the production of chemical transmitters and related topics in cellular control, and my Rockefeller University student monograph Grossberg (1964) [25] that summarized many of my discoveries since 1957, already tried to grapple with it. I concluded that rate-based, or mean value, equations could be used when the variances of the underlying stochastic processes were small compared to their means.

There were both conceptual and technological reasons to use rate-based models for many years. Conceptually, rate-based neural networks could be defined, mathematically analyzed, and computationally simulated to model how brain interactions give rise to psychological behaviors. In the final analysis, this is due to the fact the functional units of brain dynamics are spatial patterns of STM and LTM traces, not individual spikes. In addition, for many years, computers were not powerful enough to simulate even rate-based models. That is one reason why I proved so many mathematical theorems in the 1960s. I provide a historical summary in Grossberg (2026) [26] of some of these technological obstacles to theory validation.

**Figure 6:**
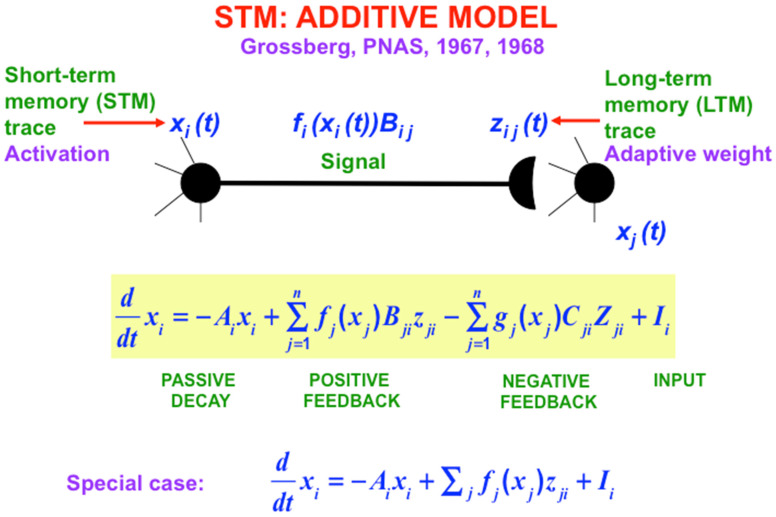
The Additive Model describes how multiple signals and inputs add up to influence the activities, or STM traces, of neurons. [Reprinted with permission from Grossberg (2021)] [2].

**Figure 7:**
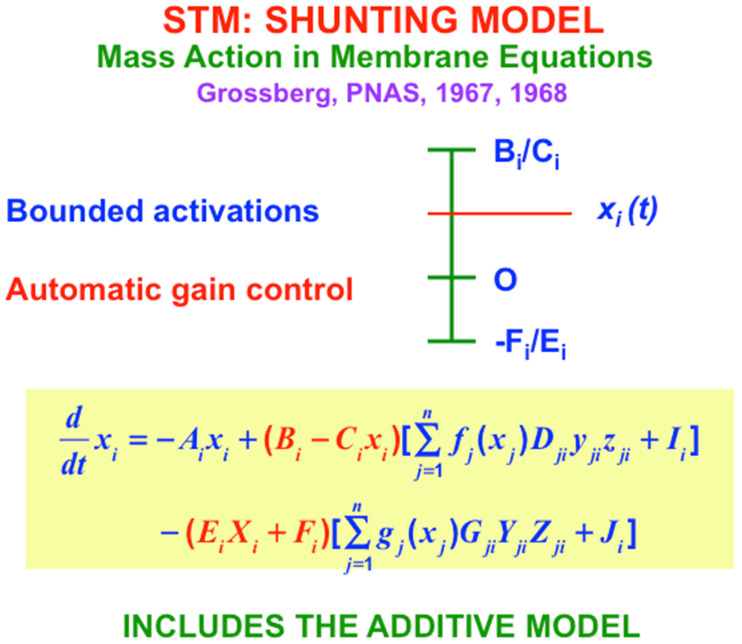
The Shunting Model includes upper and lower bounds on neuronal activities. These bounds define automatic gain control terms that multiply the additive signals and inputs, and thereby enable a shunting model to preserve its sensitivity to inputs whose size may vary greatly through time, while also approximately normalizing their total activities. [Reprinted with permission from Grossberg (2021)] [2].

**Figure 8:**
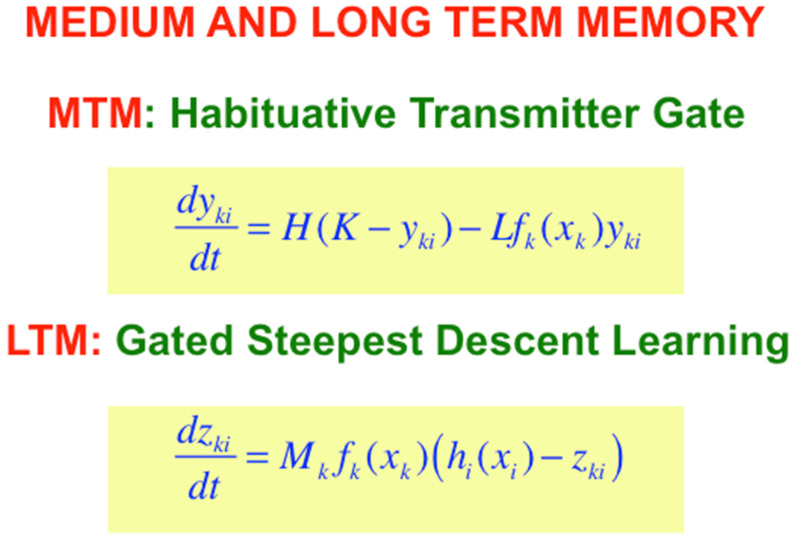
Medium-term memory (MTM) and long-term memory (LTM) equations complement the Additive and Shunting Models of STM. MTM is typically defined by a chemical transmitter that is released from the synaptic knobs of a neuron (Figure 4). Its release, or inactivation, in an activity-dependent way is also called habituation. LTM defines associative learning between a pair of neurons whose activities are correlated through time. [Reprinted with permission from Grossberg (2021)] [2].

The main equations that are used in these rate-based models are summarized in Figure 6, Figure 7 and Figure 8. I called the simplest versions of the STM equations the Additive Model (Figure 6) and the Shunting Model (Figure 7). The Additive Model got its name from the fact that each cell’s activity is the result of influences that add their effects on its rate of change. The Additive Model exhibits the problem that its responses to arbitrarily large inputs can lead to STM activities that have no upper or lower bounds, since every increment in an input can cause a proportional increment in the resulting activity. As a result, activities in an Additive Model do not saturate in response to very large inputs. In contrast, all living cells have fixed, indeed small, upper and lower bounds on their activities.

I therefore also introduced the Shunting Model, of which the Additive Model is a special case. The Shunting Model keeps cell activities within fixed upper and lower bounds (Figure 7) using *automatic gain control* terms (red terms in Figure 7) that play many useful roles in brain dynamics. But because there are fixed upper and lower bounds beyond which cell activities cannot go, the Shunting Model can also saturate its activities. Saturation of pattern activities does not happen if, as in Figure 7, excitatory, or on-center, signals are properly balanced against inhibitory, or off-surround, signals. Taken together, the excitatory and inhibitory interactions in the equation in Figure 7 define what I have called a *recurrent shunting on-center off-surround network*, or a *recurrent competitive field*, for short. Variants of this kind of network can be found throughout our brains.

One reason for their ubiquitous occurrence is that, as I first proposed in Grossberg (1973) [27], this kind of network solves a fundamental problem that I call the *noise-saturation dilemma*, which confronts all cellular systems, not only brains; namely, how can a neural network respond without saturating the activities of all its cells in response to an input pattern of increasingly large inputs, while also not losing input pattern information due to internal cellular noise when the inputs are small. This is a problem that faces all cellular systems that process distributed input patterns whose inputs can vary in size. In Grossberg (1980) [28], I derived the noise-saturation dilemma from a thought experiment that I also used to derive the shunting model from first principles.

The Shunting Model embodies the membrane equations of neurophysiology [29], which describe how cell potentials respond to their inputs. At the time, in 1957 that I derived the Shunting Model from psychological considerations, I did not yet know any neurophysiology. The thought experiment in Grossberg (1980) [28] also uses no neurophysiological information. In this sense, the Hodgkin–Huxley equations illustrate a *universal* property of all cellular systems. Variants of the Additive and Shunting models are used today by essentially all biological modelers in computational neuroscience.

With the Additive and Shunting STM equations before us, it is now easy to also write down the simplest versions of the medium-term memory, or MTM, law which is realized by a habituative transmitter gate; and the long-term memory, or LTM learning law, which is realized by gated steepest descent learning (Figure 8). MTM changes more slowly than STM, and LTM changes even more slowly than MTM. The MTM terms y_ji_ and Y_ji_ in Figure 7 are called a transmitter *gate* because they multiply STM-activated signals f_j_(x_j_) and g_j_(x_j_) that are activated by STM traces.

I have called the LTM law in Figure 8 *gated steepest descent* because learning turns on when the learning *gate*, or sampling signal, f_k_(x_k_) is positive, thereby enabling the adaptive weight, or LTM trace, z_ki_ to approach the sampled signal h_i_(x_i_) by *steepest descent*. Such a learning law enables a *pattern* of LTM traces to track a *pattern* of STM signals during learning, since an individual LTM trace can learn to become small (or large) if its STM trace also becomes small (or large).

When the learning gate shuts off, learning stops, and the adaptive weight can remember what it has learned until sampling turns on again, which might not happen for a long time. LTM also gates the read out of signals, as Figure 6 illustrates.

## 8. Why the Rate Model Is Often a Good Approximation: Spiking Frequencies

The STM activity x_j_(t) and its sampling signal f_j_(x_j_(t)) along the axon from the j^th^ cell to the i^th^ cell simplify the transformation *in vivo* from a cell body potential to a burst of spikes that travel down an axon. Term f_j_(x_j_(t)) represents the *frequency* of spikes during a short time interval around each time t. This is a plausible approximation because the response rate of a postsynaptic potential x_i_ is often slower than the rate with which spikes are emitted from a presynaptic cell. When this is true, the postsynaptic potential responds to the average number of spikes over short time intervals; that is, to the spiking frequency.

## 9. From Rate to Spiking Neurons: Hodgkin-Huxley Equations for the Squid Giant Axon

With this background in mind, I will review some biophysical models of how spikes are generated and propagate down axons of variable length without decrement to the axonal synaptic knob. Classical experimental work was done on the squid giant axon because it was big enough to allow a recording electrode to be placed inside it. Sir Alan Hodgkin and Sir Andrew Huxley did these experiments at the Physiological Laboratory in Cambridge, England, and the Laboratory of the Marine Biological Association in Plymouth, Massachusetts [29]. This work remains one of the most important neurophysiological studies that has ever been done. Hodgkin and Huxley also introduced the Hodgkin–Huxley model of nerve impulse propagation to explain their data about how spikes are generated and travel down an axon, thereby providing an inspiring example of a successful interdisciplinary experimental and modeling enterprise for which they won the Nobel Prize in Physiology or Medicine in 1963.

**Figure 9:**
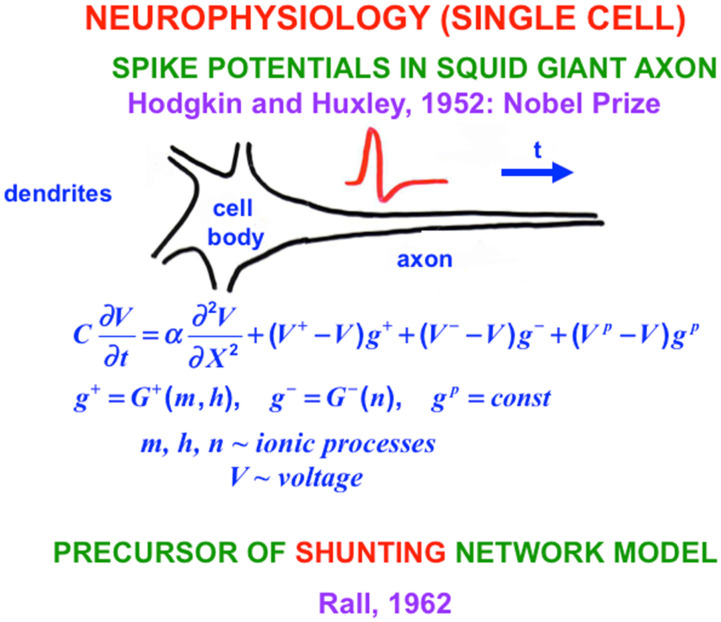
Hodgkin and Huxley developed a model to explain how spikes travel down the squid giant axon. Gail Carpenter generalized their empirical model and showed how this Generalized Hodgin-Huxley model can generate multiple types of spiking dynamics. [Reprinted with permission from Grossberg (2021)] [2].

**Figure 10:**
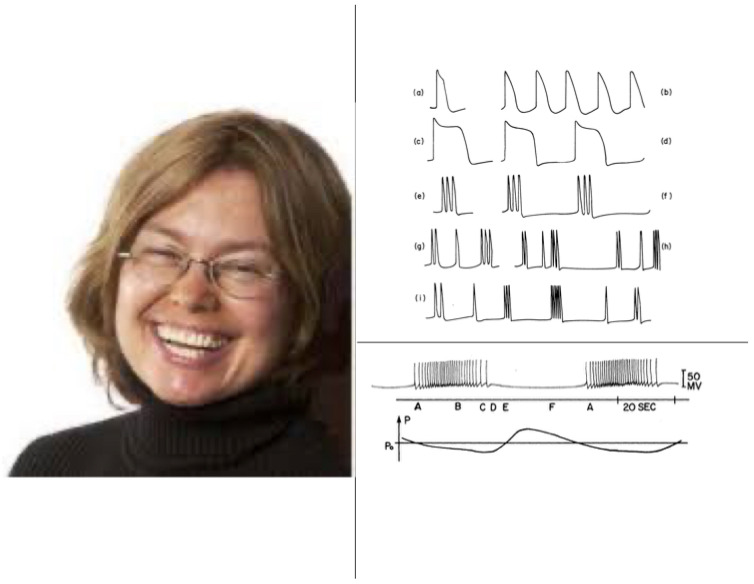
Picture of Gail Carpenter (**left**) and of some of the types of nerve impulse dynamics that Gail mathematically explained (**right**). [Reprinted with permission from Grossberg (2026)] [26].

Despite its great importance, the Hodgkin-Huxley model is a curve fit to their data from a single kind of cell. Between 1976 and 1981, Gail Carpenter introduced a Generalized Hodgkin–Huxley model that includes many biophysical variations which could occur in different kinds of neurons and species (Figure 9), as well as a mathematical analysis of how individual spikes are generated in a wide variety of cells. Carpenter also proved when and how periodic sequences and bursts of spikes could occur under both normal and abnormal conditions [30,31,32,33,34]. Her work classified different kinds of bursts of spikes, including bursts that could efficiently drive the responses of postsynaptic neurons or muscles (Figure 10). Perhaps most remarkably, she constructed a *single* Hodgkin–Huxley neuron that could generate an *arbitrary series of bursts*, including chaos. Her mathematical results provided a deep geometrical insight, using singular perturbation theory, into how and when different spiking patterns are caused in large classes of model neurons, which enabled her to design model neurons with particular spiking properties.

Gail Carpenter and I began to collaborate on neural network modeling shortly after she came to MIT as an instructor in 1974, when I was an associate professor there. One initial point of contract was that both the Hodgkin–Huxley equation for individual neurons (Figure 9) and my equations for shunting neural networks (Figure 7) used the membrane equation of neurophysiology, which I first derived in 1957 and started to get published in PNAS in 1968 (Grossberg, 1968e, equation (12)) [18]. Gail and I have now been together for over 50 years and have collaborated on many articles about a wide range of topics in neural networks.

## 10. Rate Neural Models Generate Spiking Models with Preserved Properties

As I earlier noted, the functional units of brain processing are distributed patterns of STM and LTM traces. These STM patterns can activate, and be activated by, spiking neurons. Although my colleagues and I used rate-based neural models for many years, we later developed models with spiking neurons, especially where such models could explain finer data about brain dynamics. This process generated the question: Would all those rate-based models have to be discarded and spiking models developed from scratch?

Fortunately, the answer is No! This was shown in an article with my postdoctoral fellow, Yongqiang Cao, in 2012 [35] entitled “Stereopsis and 3D surface perception by spiking neurons in laminar cortical circuits: A method of converting neural rate models into spiking models.” In this article, we showed that *all* previous articles that used the shunting equation to describe its neurons (Figure 7) could be translated into a spiking model with (at least) the same explanatory range!

Yongqiang and I had earlier, in 2005 [36], further developed my FACADE (Form-And-Color-And-DEpth) model of 3D vision and figure-ground perception [37,38,39,40,41,42,43,44,45] to explain additional data about stereopsis and 3D vision. Our 2012 result implied, far more generally, that *all* of my rate neural models, developed over several decades with 100 PhD students, postdocs, and faculty, translated directly into spiking neuron models, with no reduction of their ability to explain and simulate the same range of psychological and neurobiological data. These articles can be downloaded from sites.bu.edu/steveg. In addition, spiking models allowed us to explain and simulate finer properties of brain dynamics, now that more powerful computers were available.

## 11. From Individual Spiking Neurons to Networks and Architectures with Spiking Neurons

Quite a few of my articles with colleagues have developed spiking neural models that grew naturally out of their rate neural model precursors (e.g., Grossberg and Versace, 2008 [46]; Leveille, Versace, and Grossberg, 2010 [47]; Palma, Grossberg, and Versace, 2012 [48]; Palma, Versace, and Grossberg, 2012 [49]; Pilly and Grossberg, 2013 [50]) that model very different kinds of brain dynamics and the psychological behaviors that they generate. As noted above, all the properties that were documented in the earlier rate model of the same phenomena were preserved. In some cases, the spiking model led to modest additional properties.

For example, Pilly and Grossberg (2013) [50] notes in its Abstract that “New properties of the spiking GridPlaceMap model include the appearance of theta band modulation. The spiking model also opens a path for implementation in brain-emulating nanochips comprised of networks of noisy spiking neurons with multiple-level adaptive weights for controlling autonomous adaptive robots capable of spatial navigation.” Pilly and Grossberg (2013) [50] is a spiking neuron version of the earlier GridPlaceMap model that was published in Pilly and Grossberg (2012) [51].

When one considers the preserved properties of transforming the rate model to a spiking model, one can see the power of the method because of the sheer range of brain processes that are conserved as spiking neurons replace rate neurons. As the Abstract in Pilly and Grossberg (2013) [50] also notes [**boldface** mine]:

“Medial entorhinal grid cells and hippocampal place cells provide neural correlates of spatial representation in the brain. A place cell typically fires whenever an animal is present in one or more spatial regions, or places, of an environment. A grid cell typically fires in multiple spatial regions that form a regular hexagonal grid structure extending throughout the environment. Different grid and place cells prefer spatially offset regions, with their firing fields increasing in size along the dorsoventral axes of the medial entorhinal cortex and hippocampus. The spacing between neighboring fields for a grid cell also increases along the dorsoventral axis. This article presents **a neural model whose spiking neurons operate in a hierarchy of self-organizing maps, each obeying the same laws. This spiking GridPlaceMap model simulates how grid cells and place cells may develop. It responds to realistic rat navigational trajectories by learning grid cells with hexagonal grid firing fields of multiple spatial scales and place cells with one or more firing fields that match neurophysiological data about these cells and their development in juvenile rats. The place cells represent much larger spaces than the grid cells, which enable them to support navigational behaviors. Both self-organizing maps amplify and learn to categorize the most frequent and energetic co-occurrences of their inputs.** The current results build upon a previous rate-based model of grid and place cell learning, and thus illustrate a general method for converting rate-based adaptive neural models, without the loss of any of their analog properties, into models whose cells obey spiking dynamics.”

## 12. Gamma Oscillations with Attentive Match vs. Beta Oscillations with Mismatch Reset

**Figure 11:**
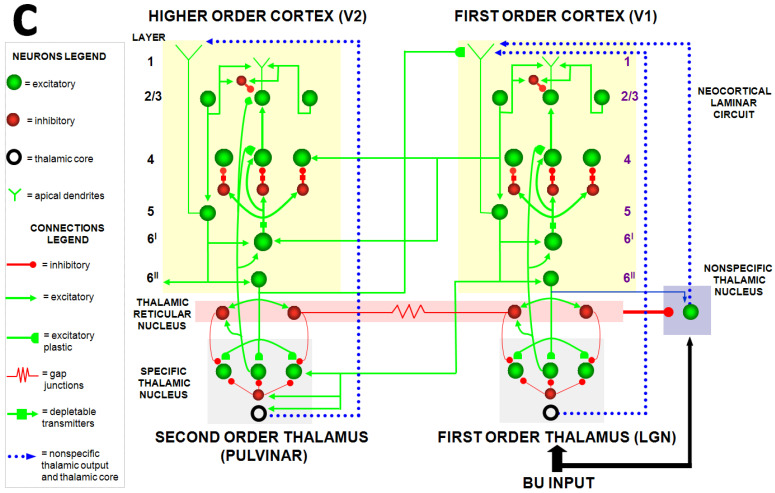
The Synchronous Matching ART, or SMART, model includes spiking neurons in a laminar cortical hierarchy. By unlumping LAMINART to include spiking neurons, finer details of neurodynamics, such as the existence of faster gamma oscillations during good enough matches, and slower beta oscillations during bad enough mismatches, could be shown as emergent properties of network interactions. [Reprinted with permission from Grossberg and Versace (2008)] [46].

Perhaps the spiking model that explains the most additional data beyond its rate model predecessors is Grossberg and Versace (2008) [46]. This Synchronous Matching Adaptive Resonance Theory (SMART) model is realized by a hierarchy of laminar cortical regions with identified anatomical, neurophysiological, biophysical, and biochemical properties (Figure 11). The abstract of this article summarizes its explanatory and predictive range [**boldface** mine]:

“This article develops the Synchronous Matching Adaptive Resonance Theory (SMART) neural model to explain how the brain may coordinate multiple levels of thalamocortical and corticocortical processing to rapidly learn, and stably remember, important information about a changing world. The model clarifies how bottom-up and top-down processes work together to realize this goal, notably how processes of learning, expectation, attention, resonance, and synchrony are coordinated. **The model hereby clarifies, for the first time, how the following levels of brain organization coexist to realize cognitive processing properties that regulate fast learning and stable memory of brain representations: single cell properties, such as spiking dynamics, spike-timing-dependent plasticity (STDP), and acetylcholine modulation; detailed laminar thalamic and cortical circuit designs and their interactions; aggregate cell recordings, such as current-source densities and local field potentials; and single cell and large-scale inter-areal oscillations in the gamma and beta frequency domains.** In particular, the model predicts how laminar circuits of multiple cortical areas interact with primary and higher-order specific thalamic nuclei and nonspecific thalamic nuclei to carry out attentive visual learning and information processing. The model simulates how synchronization of neuronal spiking occurs within and across brain regions, and triggers STDP. **Matches between bottom-up adaptively filtered input patterns and learned top-down expectations cause gamma oscillations that support attention, resonance, learning, and consciousness. Mismatches inhibit learning while causing beta oscillations during reset and hypothesis testing operations that are initiated in the deeper cortical layers.** The generality of learned recognition codes is controlled by a vigilance process mediated by acetylcholine.”

The SMART model also predicted more gamma oscillations in superficial layers of visual cortex, and beta oscillations in deeper layers. Although earlier studies had reported data consistent with gamma oscillations in response to top-down expectations that match bottom-up activity patterns [52,53,54], the beta oscillation prediction was new.

Experimental confirmations from several labs soon followed. For example, experimental support for this prediction was reported in 2011 by Elizabeth Buffalo, Pascal Fries, Rogier Landman, Timothy Buschman, and Robert Desimone [55], who found more gamma oscillations in the superficial layers of the visual cortical areas V1, V2, and V4 and more beta oscillations in deeper layers of these cortices. These authors described the slower frequencies as occurring in the alpha range, which occurs between 6–16 Hz, and which is included in the beta range that Versace and I described.

In 2009, Timothy Buschman and Earl Miller reported beta oscillations during spatial attention shifts in the frontal eye fields of monkeys [56]. Earl learned about the SMART prediction as a member of the NSF Center of Excellence for Learning in Education, Science, and Technology (CELEST) that I founded and directed at that time. Earl and Tim then reanalyzed their spatial attention data by aligning them in time with the attention shifts, reasoning that a reset would accompany every spatial attention shift. They thereby found the predicted beta oscillations.

In 2008, Joshua Berke, Vaughn Hetrick, Jason Breck, and Robert Greene reported beta oscillations during the learning of place cell selectivity while rats navigated a novel environment [57]. These results illustrate how beta oscillations may be related to ART category learning for the following reasons: It has been known since the 1990s that place cell receptive field selectivity can develop within seconds to minutes as an animal navigates, and can remain stable for months [58,59,60,61]. Place cell learning thus seems to solve the stability–plasticity dilemma. This fact raises two kinds of questions: Are these beta oscillations part of the match–mismatch gamma–beta story? And, if they are, what does top-down attention and the ART Matching Rule have to do with place cell learning? I will suggest answers to both questions in turn.

## 13. Inverted-U in Beta Power Through Time During Hippocampal Place Cell Learning

Are place cells learned using ART dynamics? The Berke et al. [57] data are consistent with this hypothesis. They showed that, paradoxically, beta power was low as a mouse traversed a lap for the first time in a novel environment, grew to full strength on the second and third laps, became low again after two minutes of exploration, and remained low on subsequent days. Beta oscillation power also correlated with the rate at which place cells became spatially selective, and did not correlate with other kinds of oscillations, notably the theta oscillations that occur during spatial navigation. Given the rapidity with which place cell learning occurred, and the sharp increase in beta activity during the second exposure to the environment, a highly selective learning mechanism was suggested.

I explained these data in a 2009 article in the following way [62]: In any ART system, the top-down adaptive weights that represent learned expectations need to be broadly distributed and large before learning occurs, so that they can match *whatever* input pattern first initiates learning of a new category. Indeed, when a new category is first activated, it is not known at the category level what pattern of features caused the category to be activated. *Whatever* feature pattern was active needs to be matched by the top-down expectation on the first learning trial, so that resonance and learning can begin. Hence the need for the *initial* values of top-down weights to be broadly distributed and sufficiently large to match *any* feature pattern. The low beta power on the first lap of exploration can be explained by this initial top-down match.

Given that the initial top-down weights are initially broadly distributed, the learning of top-down expectations is, as during all ART learning, a process of *pruning* weights on subsequent learning trials to learn to attend and match the critical feature patterns in that situation, and uses mismatch-based reset events to search for, discover, and learn new categories capable of representing the environment. Beta power on subsequent laps can be explained by mismatch reset events that correlate with the rate at which place cells become spatially selective. After learning stabilizes, there are no more mismatches, so beta power subsides.

In summary, an inverted-U in beta power through time is a signature of ART category learning in any environment.

I first heard about the Berke et al. [57] data when I gave a lecture about my new results with Versace to his department at the University of Michigan. I then had the opportunity to chat with Josh after the lecture as I met various other faculty and students who heard my talk. That is when Josh told me that he had new data about beta oscillations during the learning of new hippocampal place cells. I then did something that I rarely dare to do without first studying all the relevant published data in detail, due to the number of different factors that may influence experimental outcomes. In this case, because of the basic issues that his data may have probed, I said that, if his data have anything to do with basic properties of ART matching, then he found an inverted-U in beta power. Josh then told me, to my delight, that this was the main finding of their study.

## 14. Classifying How Signal Functions Transform Inputs into STM Traces: Sigmoids

**Figure 12:**
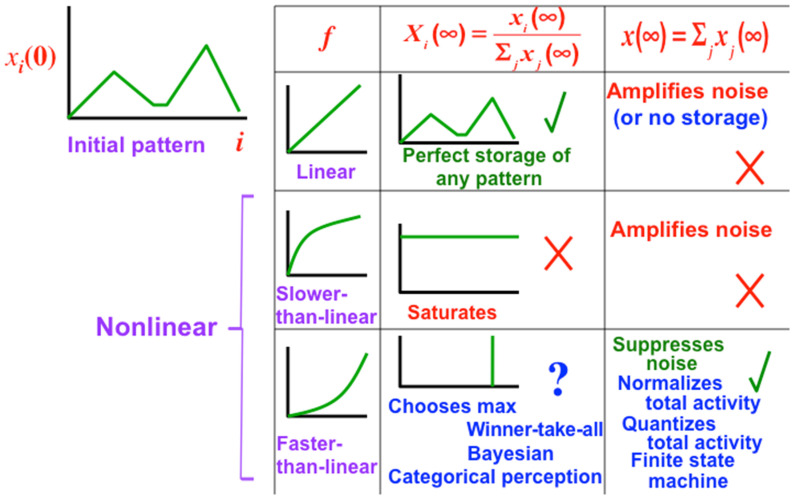
The choice of signal function f determines how an initial activity pattern is transformed and stored in short-term memory, or STM. Among linear, slower-than-linear, and faster-than-linear signal functions, only a faster-than-linear signal function can suppress noise. It does so as it chooses the population that receives the largest input for storage, while suppressing the activities of all other populations, thereby giving rise to a winner-take-all choice. [Reprinted with permission from Grossberg (2021)] [2].

**Figure 13:**
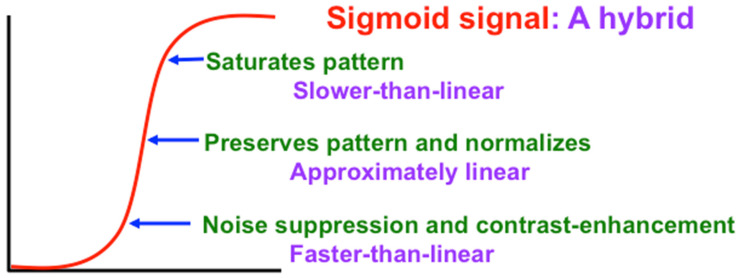
A sigmoid, or S-shaped, signal function is a hybrid signal that combines the best properties of faster-than-linear, linear, and slower-than-linear signals. It can suppress noise and store a partially contrast-enhanced activity pattern. [Reprinted with permission from Grossberg (2021)] [2].

**Figure 14:**
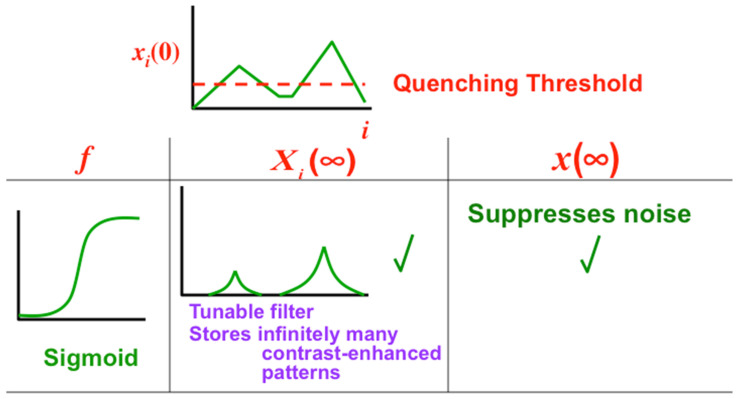
A sigmoid signal function generates a quenching threshold below which cell activities are treated like noise and suppressed. Activities that are larger than the quenching threshold are contrast enhanced and stored in short-term memory. [Reprinted with permission from Grossberg (2021)] [2].

In recurrent shunting on-center off-surround networks, such as those in Figure 7, cells communicate by converting their cell potentials x_j_ into spiking frequencies in excitatory feedback signals f_j_(x_j_) and inhibitory feedback signals g_j_(x_j_). In 1973, I began to prove theorems that classify how these feedback signal functions help to determine how the network transforms its excitatory I_i_ inputs and inhibitory J_i_ inputs into STM activities that are temporarily stored by the excitatory on-center feedback signals, balanced against inhibitory off-surround signals to prevent saturation [27]. Figure 12, Figure 13 and Figure 14 illustrate how choosing different feedback signals alters the transformation of inputs into STM traces in the simple case where all the feedback signals f_j_ and g_j_ are the same.

Figure 12 shows that Linear and Slower-than-linear signal functions both amplify noise, so cannot be used. Faster-than-linear signal functions suppress noise but do it so vigorously that they suppress all but the population that has the largest initial activity. A faster-than-linear signal function thus causes a Winner-Take-All, or WTA, choice. I believe that my 1973 article was the first one to prove this fact mathematically.

For some applications, a WTA choice is useful. However, for many applications, one wants to suppress noise, but to allow multiple populations to store STM activities in a way that reflects the initial size of their inputs.

A Sigmoid signal function has the requisite properties, as Figure 13 and Figure 14 show. That is because a Sigmoid signal is a hybrid signal function that combines the best properties of the other types of signal functions to suppress noise while contrast-enhancing and storing in STM the activities of populations whose initial activities exceed a Quenching Threshold. Activities less than the Quenching Threshold define noise. The Quenching Threshold allows the network to perform like a tunable filter using, say, a variable nonspecific arousal source to redefine initial activities that will be treated like noise due to ongoing motivational and novelty properties, among others.

## 15. How Is a Sigmoid Signal Function Constructed Within a Spiking Neural Network?

Given the importance of Sigmoid feedback signals in recurrent neural networks, a major question is: How are they constructed in a spiking neural network, where they cannot just be defined by fiat? I answered this question in a collaboration with my PhD student, Jesse Palma, and my postdoctoral fellow, Max Versace [48,49]. The Abstract of Palma, Versace, and Grossberg (2012) [49] summarizes our proposed solution of this problem [**boldface** mine]:

“Recurrent networks are ubiquitous in the brain, where they enable a diverse set of transformations during perception, cognition, emotion, and action. It has been known since the 1970s how the choice of feedback signal function can control the transformation of input patterns to rate-based recurrent on-center off-surround networks into activity patterns that are stored in short term memory. A sigmoid signal function may, in particular, control a quenching threshold below which inputs are suppressed as noise and above which they may be contrast enhanced before the resulting activity pattern is stored. The threshold and slope of the sigmoid signal function determine the degree of noise suppression and of contrast enhancement. **This article analyses how sigmoid signal functions and their shape may be determined in biophysically realistic spiking neurons. Combinations of fast, medium, and slow afterhyperpolarization (AHP) currents, and their modulation by acetylcholine (ACh), can control sigmoid signal threshold and slope. Instead of a simple gain in excitability that was previously attributed to ACh, cholinergic modulation may cause translation of the sigmoid threshold. This property clarifies how activation of ACh by basal forebrain circuits, notably the nucleus basalis of Meynert, may alter the vigilance of category learning circuits, and thus their sensitivity to predictive mismatches, thereby controlling whether learned categories code concrete or abstract information, as predicted by Adaptive Resonance Theory.”**

This research project provides an excellent example of how questions about finer neurobiological issues like the origin of sigmoid signals in spiking neurons drives one into issues that, at the time, felt like acquired tastes, notably the need to study cholinergic modulation of interacting fast, medium, and slow afterhyperpolarization currents.

## 16. How Spiking Neurons Preserve Spatial Patterns During Development: Self-Similarity

Given that spiking neurons are ubiquitous in our brains, can they preserve properties of spatial pattern learning in the child as our brains develop from childhood into adulthood? If they could not, then information learned by a child’s smaller brain would have to be relearned every time its brain increased in size during development into adulthood, or when changes in the relative distances between pairs of connected brain regions occurred.

Early steps towards a proposal for how this works were noted in Grossberg (1971) [63] where I proposed how developmentally invariant spatial pattern learning could be achieved [**boldface** mine]:

“An important constraint in our theorems is that the time lag from a given cell for signal transfer to all the cells in a functionally coordinated unit depend only on the source cell. **How can different axons from the given cell have the same time lag if they have different lengths? Clearly, then, signal velocity is proportional to axon length. But signal velocity is a local property of signal transmission, whereas axon length is a global feature of the anatomy. How can this global property be converted into a locally discernible one? A simple way is to let axon length be proportional to axon diameter, and then to let signal velocity be proportional to axon diameter.** The latter is often the case (Ruch et al., 1961) [64]. The former is qualitatively true: longer axons of a given cell type are usually thicker. Intuitively, this condition means that one idealized cell of a given type can be converted into another of the same type simply by blowing up spatial and temporal scales by a common factor; that is, **“form” is invariant under size changes. We call this property spatiotemporal self-similarity** (Grossberg, 1968e)” [18]. How can such a linear relationship between axon diameter and conduction velocity be achieved? Early neurophysiological studies reported such a linear relationship in myelinated axons that carry spiking signals via saltatory conduction between nodes of Ranvier distributed between myelinated axonal regions. If the lengths and diameters of such axons changed proportionally during development, then spatiotemporal self-similarity and spatial pattern learning and memory that is preserved under developmental changes in axon lengths would be achieved.

Early experimental and theoretical support for this proposal was reviewed in Seidl (2014) [65]: “Measurements of conduction velocity in axons attracted attention very early in the history of neuroscience. Empirical [66,67,68,69] and theoretical studies [70] showed a linear relationship between diameter and conduction velocity in myelinated axons. The myelin sheath is provided by specialized glial cells: the oligodendrocytes in the central nervous system (CNS) and Schwann cells in the peripheral nervous system (PNS). The myelin sheath is interrupted in regular intervals by the nodes of Ranvier, where sodium channels are present in high density, thereby enabling saltatory conduction [71], the basis of fast signal propagation along myelinated axons.”

## 17. Concluding Remarks

This article reviews and synthesizes highlights of the history of neural models of rate-based and spiking neural networks. It explains that theoretical and experimental results about how *all* rate-based neural network models whose cells obey the membrane equations of neurophysiology, also called shunting laws, can be converted into spiking neural network models without any loss of explanatory power, and often with gains in explanatory power. These results are relevant to all the main brain processes, including individual neurons and networks for perception, learning, cognition, and navigation. The results build upon the hypothesis that the functional units of brain processes are spatial patterns of cell activities, or short-term-memory (STM) traces, and spatial patterns of learned adaptive weights, or long-term-memory (LTM) patterns. It is also shown how spatial patterns that are learned by spiking neurons during childhood can be preserved even as the child’s brain grows and deforms while it develops towards adulthood. Indeed, this property of spatiotemporal self-similarity may be one of the most powerful properties that individual spiking neurons contribute to the development of large-scale neural networks and architectures throughout life.

## Data Availability

No new data were created or analyzed in this study.
